# The Role of Genetics in Congenital Heart Disease-Associated Pulmonary Arterial Hypertension

**DOI:** 10.1007/s00246-025-03847-z

**Published:** 2025-04-04

**Authors:** Fatma Hayvaci Canbeyli, Kazim Secgen, Fatih Suheyl Ezgu, Gulten Tacoy, Serkan Unlu, Hidayet Ozan Arabacı, Ayhan Pektas, Aslı Inci, Ergun Barıs Kaya, Umit Yasar Sinan, Mehmet Serdar Kucukoglu, Serdar Kula

**Affiliations:** 1 Division of Pediatric Cardiology, Gazi University Faculty of Medicine, Ankara, Turkey; 2 Division of Pediatric Genetics, Gazi University Faculty of Medicine, Ankara, Turkey; 3 Department of Cardiology, Gazi University Faculty of Medicine, Ankara, Turkey; 4 Department of Cardiology, Istanbul University Faculty of Medicine, Istanbul, Turkey; 5 Division of Pediatric Cardiology, Afyonkarahisar University of Health Sciences, Afyonkarahisar, Turkey; 6 Divison of Pediatric Metabolism and Nutrition, Gazi University Faculty of Medicine, Ankara, Turkey; 7 Department of Cardiology, Hacettepe University Faculty of Medicine, Ankara, Turkey

**Keywords:** Pulmonary arterial hypertension, Congenital heart disease, Genetic, Pediatric cardiology

## Abstract

Pulmonary arterial hypertension associated with congenital heart disease (APAH-CHD) is a severely progressive condition with complex pathogenesis. The aim of this study was to evaluate the contribution of genetic variants to the development of PAH in patients with APAH-CHD. Fifteen children and twenty-seven adults diagnosed with APAH-CHD were enrolled. Targeted next-generation sequencing was performed on PAH-associated genes (*ABCC8*, *ACVRL1*, *AQP1*, *ATP13A3*, *BMPR2*, *CAV1*, *GDF2*, *GGCX*, *EIF2AK4*, *ENG*, *KCNK3*, *KDR*, *KLK1*, *SMAD1*, *SMAD4*, *SMAD9*, *SOX17*, *TBX4*, *TET2*). A total of 21 distinct variants across 11 different genes were detected in 17 of the 42 patients. (*ABCC8* = 2, *ACVRL1* = 1, *ATP13A3* = 2, *BMPR2* = 4, *GGCX* = 1, *EIF2AK4* = 2, *ENG* = 1, *KDR* = 3, *SMAD1* = 1, *SMAD9* = 1, *TET2* = 3). Five of the patients with the mutation were under the age of 18, and 12 patients were adults. The most common CHD in patients with detected variants was VSD. PAH-related genetic variants were not uncommon in APAH-CHD patients. Our study identified 12 novel variants that may help to understand the genetic basis of APAH-CHD. **Trial Registration** The study has been registered on ClinicalTrials.gov with the identification number NCT05550389.

**Supplementary Information:**

The online version contains supplementary material available at 10.1007/s00246-025-03847-z.

## Introduction

Pulmonary arterial hypertension (PAH) is a rare and progressive disorder characterized by the occlusion of small pulmonary arterioles, leading to elevated pulmonary artery pressure and vascular resistance, ultimately resulting in right ventricular failure and premature death [1, 2]. Pulmonary hypertension (PH) is classified into five clinical subtypes: Group 1 PAH, Group 2 PH associated with left heart disease, Group 3 PH due to lung diseases and/or hypoxia, Group 4 PH associated with pulmonary artery obstructions, and Group 5 PH due to unclear and/or multifactorial mechanisms [3].

Despite the advances in treatment in recent years, PAH is still a fatal rare disease with high mortality and morbidity [4]. The etiology of PAH is complex and heterogeneous, involving both genetic and environmental factors. Over the past two decades, significant advances have been made in understanding the genetic underpinnings of PAH, revealing that both rare and common genetic variants contribute to disease susceptibility and progression [5–7].

PAH associated with congenital heart disease (APAH-CHD) is the main type of PAH that belongs to Group 1. There are different prevalence data regarding APAH-CHD due to differences in population studies. So the prevalence of PAH in adults with CHD remains uncertain. A study from the Netherlands showed that the prevalence of PAH in adult CHD was 3.2% [8]. While in European Congenital Heart Disease Survey Database, with a prevalence of APAH-CHD of 28% [9], it has been reported that patients with APAH-CHD account for 30.2% of PAH patients in the UK [10]. In addition, an study from USA showed that approximately 75% children with PAH had a CHD diagnosis [11].

There are different clinical profiles of APAH-CHD, such as Eisenmenger syndrome, PAH associated with systemic-to-pulmonary shunts, PAH with incidental congenital heart disease, and PAH after surgical correction. The presence of a chronic large left-to-right shunt leads to increased pulmonary blood flow and, thus, shear stress on the pulmonary endothelium. By triggering endothelial dysfunction and pulmonary vascular remodeling and obliteration of the microvasculature, the process, which is initially reversible, becomes irreversible over time. However, since PAH develops in some CHD patients and not in others, it is thought that genetic predisposition, other than the accepted pathophysiological process, may play a role in the PAH formation process in these patients [12]. Variants, including de novo changes, have been reported in genes such as *BMPR2, SOX17,* and *SMAD9* in APAH-CHD [13–15].

Many studies have been conducted on the genetic background of IPAH and HPAH, and progress has been made in this regard. However, studies in the literature on APAH-CHD are very few and the patient population is quite limited.

This publication represents the first comprehensive study to investigate the genetic characteristics of APAH-CHD patients in Turkey, offering novel insights into the genetic landscape and hereditary factors influencing PAH. We primarily aimed to identify novel genetic variants in APAH-CHD and to determine the frequency of previously detected genetic mutations. By integrating these findings with existing knowledge, we aim to enhance the understanding of the genetic basis of APAH-CHD. This research has the potential to inform future genetic screening programs and support the development of tailored management plans for PAH patients.

## Methods

### Study Population

This is a prospective, cross-sectional, and multicenter study. Fifteen children with APAH-CHD under the age of 18 and 27 adults with APAH-CHD participated in this study.

PAH was defined as mean pulmonary artery pressure (mPAP) ≥ 25 mmHg, patients diagnosed after recent guidelines with mPAP ≥ 20 mmHg, pulmonary capillary wedge pressure (PCWP) ≤ 15 mmHg, and pulmonary vascular resistance index (PVRI) ≥ 3 WU m^2^, measured by right heart catheterization [3, 16]. Patients who underwent right heart cardiac catheterization and had congenital heart disease were included. Congenital heart diseases were diagnosed by echocardiography. Patients with other diseases known to cause pulmonary hypertension, such as connective tissue disease, and those with a familial history of PAH were excluded from the study.

Differences between mutation carriers and non-carriers in terms of age at diagnosis, 6 min walking test (6MWT), World Health Organization functional class (WHO FC), hemodynamic parameters (mean pulmonary arterial pressure (mPAP), right atrial pressure (RAP), pulmonary vascular resistance (PVR), PVR/SVR), and combined therapy were evaluated.

This study has been approved by the ethics committee of Gazi University (approval date: 12.23.2019, Ref. Number: 24074710-604.01.01-01) and adheres to the highest research standards. Informed consent was obtained from all participants. The study has been registered on ClinicalTrials.gov with the identification number NCT05550389.

### Gene Panel Design and Selection

A targeted next-generation sequencing (NGS) panel was developed to screen for PAH-associated genes that have been previously implicated in PAH. The 19 genes included in this panel were selected based on a combination of factors, including prior evidence of strong disease associations, mutation frequency in PAH cohorts, and their role in key biological pathways relevant to PAH pathogenesis.

The selection process prioritized genes known to be involved in pulmonary vascular remodeling, endothelial dysfunction, and TGF-β/BMP signaling, which are major mechanisms contributing to PAH. Genes such as *BMPR2*, *ACVRL1*, *SMAD1*, *SMAD4*, and *SMAD9* were included due to their established role in the BMP/TGF-β signaling pathway, which regulates pulmonary vascular homeostasis and has been extensively linked to both heritable and idiopathic PAH. *EIF2AK4* was included due to its association with pulmonary veno-occlusive disease (PVOD), a severe PAH subtype.

Genes involved in vascular endothelial function and angiogenesis, such as *KDR, ENG*, and *GDF2*, were selected based on their role in vascular integrity, endothelial proliferation, and response to hypoxia, all of which are critical in PAH pathophysiology. *ATP13A3* and *ABCC8*, which are involved in ion transport and cellular metabolism, were included due to emerging evidence suggesting their contribution to PAH through dysregulation of pulmonary vascular tone.

Additionally, *SOX17*, *TBX4*, and *TET2* were included due to their reported association with developmental vascular anomalies, congenital heart disease (CHD), and hematopoietic regulation, all of which may contribute to the development of APAH-CHD. The final panel composition was guided by previous studies, functional significance, and mutation frequency in PAH patient registries.

### Sample Collection and DNA Extraction

Peripheral blood samples were collected from all participants, and DNA was extracted from peripheral leukocytes using the Iprep™ Purelink gDNA Blood Kit (Invitrogen, Carlsbad, CA) according to the manufacturer's protocol. The quality and quantity of extracted DNA were assessed using standard methods.

### Targeted Next-Generation Sequencing

The QIAseq Human Exome Kit (Qiagen, USA) was used to target coding exons and flanking intronic sequences, following the manufacturer's handbook protocols. Sequencing was performed on the Illumina NovaSeq 6000 System (Illumina, San Diego, CA). DNA variants were called using QIAGEN CLC Genomics Workbench (Qiagen, USA) and aligned to the human reference genome (Hg19). Variants were annotated using QIAGEN Clinical Insight (QCI) Interpreter (Qiagen, Redwood City, CA, USA).

### In Silico Variant Analysis and Interpretation

The identified variants were analyzed using several in silico prediction tools, including SIFT, CADD, PolyPhen-2, and MutationTaster. SIFT scores < 0.05 were considered deleterious, while higher scores suggested tolerance. CADD scores ≥ 15 indicated a higher likelihood of pathogenicity. These scores were interpreted in conjunction with the clinical presentation, gene function, and relevant literature. Variants with lower SIFT and CADD scores were not excluded, as their contribution to PAH might involve mechanisms not fully captured by these prediction tools.

To further assess the clinical relevance of the variants, population frequencies were evaluated using the Genome Aggregation Database (gnomAD). Variants were classified according to the guidelines of the American College of Medical Genetics and Genomics (ACMG) [17] into categories including pathogenic, likely pathogenic, and variants of uncertain significance (VUS). Variants deemed likely benign or benign were excluded from the final interpretation. A comprehensive literature review was conducted, and the ClinVar database was queried (as of September 2024) to determine whether each variant had been previously reported. Variants absent from the literature or not listed in ClinVar—or listed without supporting clinical or functional evidence—were classified as novel. The SIFT and CADD scores, along with variant characteristics, are presented in Table 1.

**Table 1:** Summary of genetic variants identified in APAH-CHD patients and their molecular characteristics

PN	Gene	Variant type	Location	Nucleotide change	Amino acid change	RefSeq transcript	Zygosity	ACMG classification	ClinVar VID	gnomAD (exome frequency)	SIFT score	CADD score
P1	*GGCX*	Missense	Exon 2	c.137C>T	p.Ser46Phe	NM_000821.7	Het	VUS	337279	0^c^	0.04 D	25.40
	*TET2*	Stop-loss	Exon 11	c.3496T>A^a^	p.*1166Lysext*12	NM_017628.4	Het	VUS	–	0.00008062	–	< 10
P2	*ACVRL1*	Missense	Exon 3	c.140G>A^a,d^	p.Arg47Gln	NM_000020.3	Het	LP	2643006^d^	0.00001818	0.67 T	13.87
P3	*ATP13A3*	Missense	Exon 26	c.3298C>A^a^	p.Pro1100Thr	NM_001367549.1	Het	VUS	–	0^c^	0.01 D	29.50
	*ABCC8*	Missense	Exon 24	c.2929G>A	p.Ala977Thr	NM_000352.6	Het	VUS	1339208	0.00003185	0.38 T	21.90
P4	*BMPR2*	Missense	Exon 3	c.440G>A	p.Arg147Gln	NM_001204.7	Het	VUS	2374491	0.00008360	0.34 T	22.40
P5	*SMAD9*	Missense	Exon 5	c.602C>T^a^	p.Pro201Leu	NM_001127217.3	Het	VUS	–	0.00003183	0.08 T	28.10
P6	*KDR*	Missense	Exon 15	c.2270C>G^a,d^	p.Ala757Gly	NM_002253.3	Het	VUS	2348108^d^	0.00004132	0.04 D	23.80
P7	*KDR*	Intronic	Near exon 10	c.1413-3T>C^a^	–	NM_002253.3	Het	VUS	–	0^c^	–	< 10
P8	*TET2*	Missense	Exon 11	c.5978G>A^a^	p.Arg1993Gln	NM_001127208.3	Het	VUS	–	0.000006396	0.36 T	22.70
P9	*BMPR2*	Nonsense	Exon 12	c.2789C>G	p.Ser930*	NM_001204.7	Het	P	426007	0^c^	–	41
P10		Nonsense	Exon 41	c.4669C>T	p.Arg1557*	NM_001013703.4	Homo	P	1431295	0.00001203	–	49
P11	*ATP13A3*	Missense	Exon 9	c.793G>A^a^	p.Ala265Thr	NM_001367549.1	Het	VUS	–	0^c^	0.19 T	22.20
P12	*ABCC8*	Missense	Exon 19	c.2354A>C^a^	p.Glu785Ala	NM_000352.6	Het	LP	–	0^c^	0.05 D	29.90
	*KDR*	Missense	Exon 10	c.1362T>A	p.His454Gln	NM_002253.3	Het	VUS	2227055	0.0001513	0.59 T	< 10
	*TET2*	Missense	Exon 3	c.1789T>C^a^	p.Ser597Pro	NM_001127208.3	Het	VUS	–	0.00007962	0.14 T	< 10
P13	*BMPR2*	Missense	Exon 12	c.1766A>G	p.Tyr589Cys	NM_001204.7	Het	VUS	425966	0.0001949	0.03 D	27.90
P14	*ENG*	Indel	Exon 2	c.321_322delinsTT	p.His108Tyr	NM_001114753.3	Het	VUS	407139	0^c^	0.09 T	10.20
P1	*SMAD1*	Missense	Exon 4	c.425G>C^a^	p.Arg142Thr	NM_005900.3	Het	VUS	–	0^c^	0.13 T	25.20
P16	*BMPR2*	Missense	Exon 12	c.2677C>T^a^	p.Arg893Trp	NM_001204.7	Het	VUS	–	0.000002920	0.02 D	25.30
P17	*EIF2AK4*	Missense	Exon 35	c.4115T>C	p.Ile1372Thr	NM_001013703.4	Het	VUS	1418081	0.0001833	0.02 D	24.10

*PN* patient number, *Homo* homozygous, *Het* heterozygous, *P* pathogenic, *LP* likely pathogenic, *VUS* variant of uncertain significance

Clin Variation ID (ClinVar VID D: A unique identifier assigned to variants submitted to the ClinVar database, which provides information on the clinical significance of genetic variants)

*gnomAD* Genome aggregation database, *CADD* Combined Annotation Dependent Depletion, *SIFT* Sort Intolerant from Tolerant

^a^Novel variants were defined as those not previously reported in the literature or not listed in the ClinVar database with supporting clinical or functional evidence, as of September 2024

^b^gnomAD frequencies are based on data available as of September 2024

^c^This variant is not listed in the gnomAD exomes database, although its locus is covered in gnomAD exomes

^d^This variant has been submitted once to ClinVar as a VUS, with no associated clinical or functional data. To our knowledge, it has not been previously reported in the scientific literature and is therefore considered novel in this context

### Statistical Analysis

All statistical analyses were conducted using SPSS software (version 22, IBM, Chicago, IL, USA). Continuous variables were presented as mean ± standard deviation or median (range) according to the normality tests and compared using a two-sided Student’s *t* test or Mann–Whitney *U* test, as appropriate. Categorical variables were compared using Chi square test. The statistical significance was set at *p* value < 0.05.

## Results

**Fig. 1:**
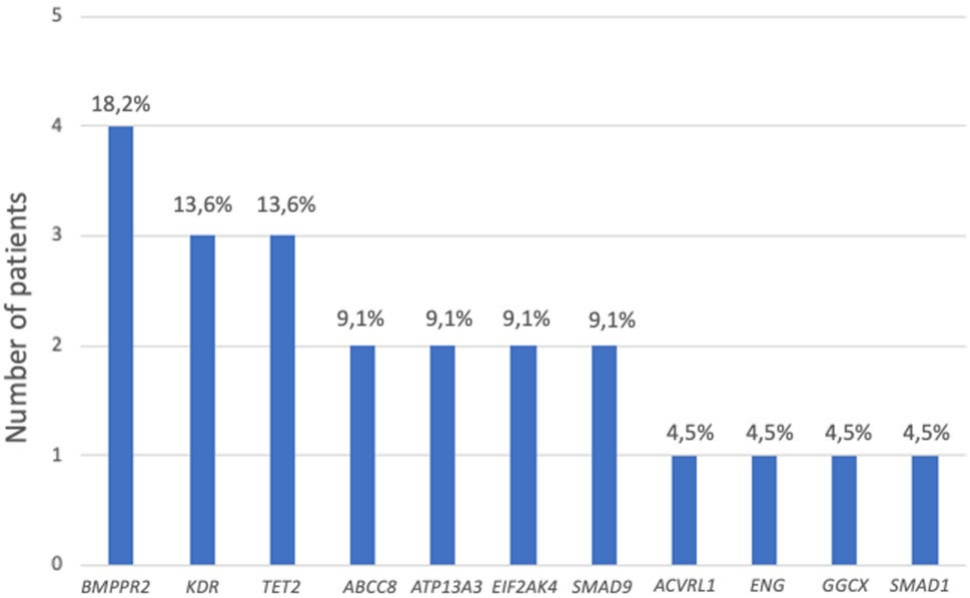
Distribution of variants by gene

Genetic analysis identified 21 distinct variants across 11 different genes in 17 of the 42 patients (Table 1, Fig. 1). Among these, 12 variants were classified as novel, not previously reported in the literature or listed in ClinVar with supporting clinical or functional evidence (as of September 2024). The detected variants included 16 missense, two nonsense, one indel, one stop-loss, and one intronic variant, reflecting the genetic heterogeneity of APAH-CHD.

The most frequently affected genes in our cohort were *BMPR2, KDR, TET2,* and *EIF2AK4* (Fig. 1). *BMPR2* variants were detected in four patients, including the pathogenic nonsense variant (c.2789C>G, p.Ser930*) and one novel missense variant (c.2677C>T, p.Arg893Trp), all in patients with VSD. Notably, three of the *TET2* variants and two of the *KDR* variants were novel, underscoring the potential contribution of rare or previously unrecognized variation in these genes to APAH-CHD. *EIF2AK4* variants were identified in two patients, one of whom carried a homozygous pathogenic variant (c.4669C>T, p.Arg1557*). The remaining variants were distributed across *ABCC8, ACVRL1, ATP13A3, ENG, GGCX, SMAD1*, and *SMAD9,* six of which were identified as novel (Table 1). One novel *ACVRL1* variant (c.140G>A, p.Arg47Gln) and one novel *ABCC8* variant (c.2354A>C, p.Glu785Ala) were classified as likely pathogenic. Although the variants were classified as pathogenic or likely pathogenic, the patients carrying them differed in disease severity and age at diagnosis, preventing any direct inference of clinical correlation. Among the *ABCC8* variants, one co-occurred with a novel *ATP13A3* variant, and the other with *KDR* and *TET2* variants. The high proportion of variants of uncertain significance (17/21; 81%) highlights the complexity of interpreting genetic findings in APAH-CHD.

The demographic and clinical characteristics of patients carrying variants are summarized in Table 2. Among the 17 patients with genetic variants, five were under the age of 18, while 12 were adults. The most common congenital heart defect among variant carriers was ventricular septal defect (VSD). Four patients in the cohort had Down syndrome, and among them, three carried at least one identified genetic variant. None of the patients with detected variants had a positive vasoreactivity test.

**Table 2:** Clinical characteristics of patients with identified variants

Patients	Gene	Congenital heart diseases	Gender	Age/years	PAH diagnosis age	WHO FC	Treatment	mPAP (mmHg)	mRAP (mmHg)	PVR (WU)	PVR/SVR
P1	*GGCX*	VSD	Male	35	15	II	Bosentan, sildenafil, tadalafil	38	8	9	0.46
	*TET2*										
P2	*ACVRL1*	VSD, secundum ASD	Male	15	3	II	Bosentan, iloprost	43	4	21	0.46
P3	*ATP13A3*	AVSD	Male	16	8	II	Bosentan, sildenafil	85	6	21	2.1
	*ABCC8*										
P4	*BMPR2*	VSD	Female	12	1	II	Sildenafil	26	4	5	0.29
P5	*SMAD9*	secundum ASD	Female	51	41	II	Tadalafil, selexipag	38	10	5	0.30
P6	*KDR*	VSD	Female	46	33	II	Bosentan, tadalafil	80	8	12	0.73
P7	*KDR*	AP window	Female	41	7	II	Bosentan, sildenafil, tadalafil	84	7	10	0.33
P8	*TET2*	TGA, VSD	Female	15	3	III	Bosentan, sildenafil	82	4	11	0.55
P9	*BMPR2*	VSD	Female	42	16	III	Bosentan, tadalafil	56	9	14	0.66
P10	*EIF2AK4*	VSD	Female	58	38	II	Bosentan, tadalafil	48	8	12	0.60
P11	*ATP13A3*	DORV	Male	19	1	III	Macitentan	36	5	9	0.52
P12	*ABCC8*	secundum ASD	Male	76	70	III	Macitentan	28	18	4	0.30
	*KDR*										
	*TET2*										
P13	*BMPR2*	VSD	Female	56	44	II	Bosentan, sildenafil, selexipag	42	8	6	0.33
P14	*ENG*	VSD, secundum ASD	Male	5	1	III	Bosentan, sildenafil	48	15	9	0.39
P15	*SMAD1*	VSD	Female	18	5	II	Bosentan	96	5	18	0.66
P16	*BMPR2*	VSD	Male	45	28	II	Bosentan, tadalafil	56	8	9	0.45
P17	*EIF2AK4*	Secundum ASD, LPA hypoplasia	Female	19	14	II	Bosentan	29	4	3	0.21

*VSD* ventricular septal defect;* ASD* atrial septal defect, *AP window* aortopulmonary window, *TGA* transposition of the great arteries, *DORV* double outlet right ventricle, *LPA* left pulmonary artery, *mPAP* mean pulmonary artery pressure, *mRAP* mean right arterial pressure, *PVR* pulmonary vascular resistance; *SVR* systemic pulmonary resistance, *WU* wood units, *WHO-FC* World Health Organization functional class

Clinical and hemodynamic characteristics at the time of diagnosis were evaluated in mutation carriers and non-carriers. No statistical difference was found between the two groups in terms of age at diagnosis, gender, WHO FC, 6MWT and hemodynamic parameters, and combined treatment or monotherapy (Table 3).

**Table 3:** Clinical and hemodynamic parameters of mutation carriers and non-carriers

	Mutation carriers n:17	Non-carriers n:25	*P* value
Age (years)	35 (5–76)	28 (12–75)	0.74
Age at diagnosis (years)	14 (1–70)	13 (2–69)	0.36
Sex M/F	11/6	15/10	0.75
WHO FC			
I		1	0.28
II	12	21	
III	5	3	
IV			
6MWT (m)	429.14 ± 74.19	464.08 ± 103.31	0.27
mPAP (mmHg)	52.8 ± 22.8	48.4 ± 18.6	0.49
mRAP (mmHg)	7 (4–18)	8 (3–18)	0.67
PVR (WU)	9 (2.4–21)	6.4 (1.3–31.9)	0.13
PVR/SVR	0.46 (0.2–2.1)	0.48 (0.1–1.9)	0.65
Treatment			
Monotherapy	4	8	0.73
Combined therapy	13	17	

*WHO-FC* World Health Organization functional class, *mPAP* mean pulmonary artery pressure, *mRAP* mean right arterial pressure, *PVR* pulmonary vascular resistance, *SVR* systemic pulmonary resistance, *WU* wood units

## Discussion

The genetic architecture of PAH is notably heterogeneous, with contributions from multiple genes. This heterogeneity underscores the need for comprehensive genetic screening to capture the full spectrum of genetic contributors [18]. Our study aimed to elucidate the genetic etiology in patients with APAH-CHD by screening 19 genes previously associated with PAH. This is the first comprehensive investigation of the genetic characteristics of APAH-CHD patients in Turkey. Given the rarity of this condition and limited global research, our study holds a distinctive position. While progress has been made in understanding PAH genetics, few studies have specifically focused on congenital heart disease patients. This study is among the largest studies that have examined the genetic characteristics of APAH-CHD patients, further underscoring its significance.

Variants in the *BMPR2* gene represent the most frequent genetic cause of PAH, identified in approximately 29% of familial and idiopathic cases [7, 19], and are also common in APAH-CHD cohorts [18]. We detected *BMPR2* variants in 18% of our APAH-CHD cohort (Fig. 1), consistent with the literature, including a pathogenic nonsense variant (c.2789C>G, p.Ser930*) and a novel missense variant (c.2677C>T, p.Arg893Trp) (Table 1). Both variants likely disrupt BMP signaling, which is crucial for pulmonary vascular homeostasis. All *BMPR2* variant carriers had VSD (Table 2), but our sample size was small to assess the association of the mutation with CHD.

While in the literature, variants in *EIF2AK4* are prominently associated with pulmonary veno-occlusive disease (PVOD), a severe PAH subtype characterized by distinctive clinical and pathological features [20], our patients showed no signs of PVOD. One patient exhibited a homozygous pathogenic variant in *EIF2AK4*, whereas another patient harbored a heterozygous variant of uncertain clinical significance (Tables 1 and 2). Variants in the *KDR* gene, alongside three novel *TET2* variants, were among the most commonly identified in our cohort (Fig. 1 and Table 1), reinforcing their potential biological relevance, and two of the three *KDR* variants were novel. *KDR* encodes VEGFR2, a central receptor in VEGF signaling that regulates endothelial proliferation and vascular integrity—key processes disrupted in PAH [21]. The mPAP values of patients with *KDR* and *TET2* mutations were quite high and combined treatment was started, which may be important in determining the clinical severity in these mutations carriers (Table 2).

We identified a novel and likely pathogenic *ACVRL1* c.140G>A (p.Arg47Gln) variant in a pediatric patient with ASD secundum and VSD, diagnosed with PAH at age 3 (Tables 1 and 2). While *ACVRL1* variants are primarily associated with hereditary hemorrhagic telangiectasia (HHT) and severe pediatric PAH [22–24], our patient showed no signs of HHT and responded well to combined treatment, highlighting the need for further research into *ACVRL1* variants beyond their established HHT-related manifestations.

Novel variants in the *SMAD1* and *SMAD9* genes, both crucial downstream mediators in BMP signaling, were also identified (Table 1). Due to their roles in pulmonary vascular remodeling and cellular proliferation [25, 26], these variants represent important biological candidates for functional validation to further define their involvement in APAH-CHD.

Another notable observation was the concurrent presence of variants in *GGCX* and *TET2* within the same patient (Table 1). *GGCX* is involved in gamma-carboxylation essential for vascular function [27], and *TET2* variants have been implicated in PAH via inflammation and vascular remodeling [28]. Similarly, *ABCC8* (encoding the SUR1 subunit of ATP-sensitive potassium channels involved in vascular tone regulation) [27, 29, 30] variants were identified in two patients—one with a novel variant alongside *KDR* and *TET2* variants, and another with a novel heterozygous *ATP13A3* variant (Table 1), which is associated with ion transport dysfunction [31, 32]. These combined findings reinforce the idea that APAH-CHD may result from complex interactions among multiple genetic pathways, emphasizing the clinical importance of comprehensive genetic analyses.

Interestingly, we did not detect variants in genes commonly reported in PAH cohorts from other populations, such as *SOX17*, *TBX4*, or *KCNK3* [5, 27, 33, 34] (Fig. 1 and Table 1). The absence of these mutations may be related to geographical genetic heterogeneity, population-specific variant distributions, or the relatively small size of our cohort. Geographic variation highlights the necessity for population-specific genetic investigations to better understand local patterns of PAH-associated variants and to facilitate tailored diagnostic and therapeutic approaches.

The prevalence of developing PH in children with Down syndrome is as high as 28% [35]. Patients with CHD and Down syndrome are at an increased risk of developing Eisenmenger syndrome [3]. In our study, four patients had Down syndrome; we detected mutations in three of these patients (P3, P4, and P9). The relationship between Down syndrome and PAH is quite complex [36], and genetic mutations may also contribute to the development of PAH in Down syndrome patients with CHD. Detected genetic mutations may also be determinants in the prognosis of the disease. It has been shown that in *BMPR2* mutation carriers, PAH occurs earlier than in non-carriers with a more severe hemodynamic compromise at the time of diagnosis [37]. On the contrary, in our study, it was observed that there was no significant difference in hemodynamic findings at the time of diagnosis and WHO FC, 6MWT, which are markers used to determine the course of the disease, between mutation carriers and non-carriers. This may be related to the small sample size. In addition, genetic mutation diversity may make it difficult to evaluate the clinical effects of mutations.

There are several limitations in this study. First, our study planned to identify genetic variants in APAH-CHD patients. Due to the small sample size and the diversity of detected mutations, evaluation could not be made among the variants. Second, we could not evaluate the relationship between CHD and variants. Finally, there was no significant difference in clinical and hemodynamic parameters between mutation carriers and non-carriers. But we believe that the small sample size might affect the generalizability.

## Conclusion

Despite some advancements in understanding the genetic mechanisms behind IPAH and HPAH, there are still many uncertainties, particularly concerning the genetic factors involved in APAH-CHD. Given the complex nature of PAH, genetic variants may contribute to the development of APAH-CHD. Our study's identification of 12 novel variants aligns with the literature, highlighting the discovery of new genetic variants in PAH cohorts. The identification of these novel variants underscores the complexity of the genetic underpinnings of PAH and the necessity for comprehensive genomic screening in affected patients. Implementing genetic screening, especially for high-risk individuals, may facilitate earlier diagnosis and better treatment planning. The limited existing research on this topic highlights the need for larger studies and functional analyses to enhance our understanding of the genetic factors at play and to improve personalized care for APAH-CHD patients.

## Supplementary Information

Below is the link to the electronic supplementary material.

Supplementary file1 (DOCX 58 KB)

## Data Availability

No datasets were generated or analysed during the current study.
