# Supplementary material for: The Role of Genetics in Congenital Heart Disease-Associated Pulmonary Arterial Hypertension
Source: Pediatr Cardiol. 2025 Apr 4;47(2):821–9. doi: 10.1007/s00246-025-03847-z (PMC12855241; doi:10.1007/s00246-025-03847-z)
Supplement: Supplementary file 1 — Supplementary file1 (DOCX 58 KB) [file 246_2025_3847_MOESM1_ESM.docx]

| Gene | Mode of inheritance | Associated phenotypes | References |
| --- | --- | --- | --- |
| *ABCC8* | AD | Diabetes mellitus, noninsulin-dependent (OMIM:125853); Diabetes mellitus, permanent neonatal 3, with or without neurologic features (OMIM:618857); Diabetes mellitus, transient neonatal 2 (OMIM:610374); Hyperinsulinemic hypoglycemia, familial, 1 (OMIM:256450); Hypoglycemia of infancy, leucine-sensitive (OMIM:240800) | [1] |
| *ACVRL1* | AD | Telangiectasia, hereditary hemorrhagic, type 2 (OMIM:600376) | [2] |
| *AQP1* | AD | [Aquaporin-1 deficiency] (OMIM:110450); [Blood group, Colton] (OMIM:110450) | [3] |
| *ATP13A3* | AR | Pulmonary hypertension, primary, 5 (OMIM:265400) | [4] |
| *BMPR2* | AD | Pulmonary hypertension, familial primary, 1, with or without HHT (OMIM:178600); Pulmonary hypertension, primary, fenfluramine or dexfenfluramine-associated (OMIM:178600); Pulmonary venoocclusive disease 1 (OMIM:265450) | [5] |
| *CAV1* | AD | Lipodystrophy, congenital generalized, type 3 (OMIM:612526); Lipodystrophy, familial partial, type 7 (OMIM:606721); Pulmonary hypertension, primary, 3 (OMIM:615343) | [6] |
| *GDF2* | AD | Telangiectasia, hereditary hemorrhagic, type 5 (OMIM:615506) | [7] |
| *GGCX* | AR | Pseudoxanthoma elasticum-like disorder with multiple coagulation factor deficiency (OMIM:610842); Vitamin K-dependent clotting factors, combined deficiency of, 1 (OMIM:277450) | [8] |
| *EIF2AK4* | AR | Pulmonary venoocclusive disease 2 (OMIM:234810) | [9] |
| *ENG* | AD | Telangiectasia, hereditary hemorrhagic, type 1 (OMIM:187300) | [10] |
| *KCNK3* | AD | Pulmonary hypertension, primary, 4 (OMIM:615344) | [11] |
| *KDR* | AD | (Hemangioma, capillary infantile, susceptibility to (OMIM:602089); Hemangioma, capillary infantile, somatic (OMIM:602089) | [12] |
| *KLK1* | - | [Kallikrein, decreased urinary activity of] (OMIM:615953) | [8] |
| *SMAD1* | AD | -^*^ | [13] |
| *SMAD4* | AD | Juvenile polyposis/hereditary hemorrhagic telangiectasia syndrome (OMIM:175050); Myhre syndrome (OMIM:139210); Pancreatic cancer, somatic (OMIM:260350); Polyposis, juvenile intestinal (OMIM:174900) | [13] |
| *SMAD9* | AD | Pulmonary hypertension, primary, 2 (OMIM:615342) | [13] |
| *SOX17* | AD | Vesicoureteral reflux 3 (OMIM:613674) | [14] |
| *TBX4* | AD/AR | Amelia, posterior, with pelvic and pulmonary hypoplasia syndrome (OMIM:601360), Ischiocoxopodopatellar syndrome with or without pulmonary arterial hypertension (OMIM:147891) | [30] |
| *TET2* | - | Immunodeficiency 75 (OMIM:619126), Myelodysplastic syndrome, somatic (OMIM:614286) | [31] |
| OMIM: Online Mendelian Inheritance of Man, AD: Autosomal dominant, AR: Autosomal recessive  *: Not currently associated with any documented phenotype in the OMIM database. | | | |

**Table 1:** List of analyzed PAH-associated genes

**References**

1. Bohnen, M.S., et al., *Loss-of-Function ABCC8 Mutations in Pulmonary Arterial Hypertension.* Circ Genom Precis Med, 2018. **11**(10): p. e002087.

2. Girerd, B., et al., *Clinical outcomes of pulmonary arterial hypertension in patients carrying an ACVRL1 (ALK1) mutation.* Am J Respir Crit Care Med, 2010. **181**(8): p. 851-61.

3. Graf, S., et al., *Identification of rare sequence variation underlying heritable pulmonary arterial hypertension.* Nat Commun, 2018. **9**(1): p. 1416.

4. Machado, R.D., et al., *Biallelic variants of ATP13A3 cause dose-dependent childhood-onset pulmonary arterial hypertension characterised by extreme morbidity and mortality.* J Med Genet, 2022. **59**(9): p. 906-911.

5. Evans, J.D., et al., *BMPR2 mutations and survival in pulmonary arterial hypertension: an individual participant data meta-analysis.* Lancet Respir Med, 2016. **4**(2): p. 129-37.

6. Tomita, S., et al., *The Cavin-1/Caveolin-1 interaction attenuates BMP/Smad signaling in pulmonary hypertension by interfering with BMPR2/Caveolin-1 binding.* Commun Biol, 2024. **7**(1): p. 40.

7. Hodgson, J., et al., *Characterization of GDF2 Mutations and Levels of BMP9 and BMP10 in Pulmonary Arterial Hypertension.* Am J Respir Crit Care Med, 2020. **201**(5): p. 575-585.

8. Zhu, N., et al., *Novel risk genes and mechanisms implicated by exome sequencing of 2572 individuals with pulmonary arterial hypertension.* Genome Med, 2019. **11**(1): p. 69.

9. Eyries, M., et al., *EIF2AK4 mutations cause pulmonary veno-occlusive disease, a recessive form of pulmonary hypertension.* Nat Genet, 2014. **46**(1): p. 65-9.

10. Uznanska-Loch, B., et al., *Genetic variants in a Polish population of patients with pulmonary arterial hypertension: sequencing of BMPR2, ALK1, and ENG genes.* Kardiol Pol, 2018. **76**(5): p. 852-859.

11. Lambert, M., et al., *Characterization of Kcnk3-Mutated Rat, a Novel Model of Pulmonary Hypertension.* Circ Res, 2019. **125**(7): p. 678-695.

12. Swietlik, E.M., et al., *Bayesian Inference Associates Rare KDR Variants with Specific Phenotypes in Pulmonary Arterial Hypertension.* Circ Genom Precis Med, 2020. **14**(1): p. e003155.

13. Nasim, M.T., et al., *Molecular genetic characterization of SMAD signaling molecules in pulmonary arterial hypertension.* Hum Mutat, 2011. **32**(12): p. 1385-9.

14. Rhodes, C.J., et al., *Genetic determinants of risk in pulmonary arterial hypertension: international genome-wide association studies and meta-analysis.* Lancet Respir Med, 2019. **7**(3): p. 227-238.
